# Gene Doping with Peroxisome-Proliferator-Activated Receptor Beta/Delta Agonists Alters Immunity but Exercise Training Mitigates the Detection of Effects in Blood Samples

**DOI:** 10.3390/ijms222111497

**Published:** 2021-10-25

**Authors:** Brigitte Sibille, Isabelle Mothe-Satney, Gwenaëlle Le Menn, Doriane Lepouse, Sébastien Le Garf, Elodie Baudoin, Joseph Murdaca, Claudine Moratal, Noura Lamghari, Giulia Chinetti, Jaap G. Neels, Anne-Sophie Rousseau

**Affiliations:** 1 INSERM, Université Côte d’Azur, C3M, 06204 Nice, France; Brigitte.SIBILLE@univ-cotedazur.fr (B.S.); Isabelle.SATNEY@univ-cotedazur.fr (I.M.-S.); gwenaelle.lemenn@gmail.com (G.L.M.); dlepouse@gmail.com (D.L.); seb.legarf@hotmail.fr (S.L.G.); elodiebaudoin68@gmail.com (E.B.); joseph.murdaca@univ-cotedazur.fr (J.M.); Claudine.MORATAL@univ-cotedazur.fr (C.M.); nouritalamghari@gmail.com (N.L.); Anne-Sophie.ROUSSEAU@univ-cotedazur.fr (A.-S.R.); 2 CHU, INSERM, Université Côte d’Azur, C3M, 06204 Nice, France; Giulia.CHINETTI@univ-cotedazur.fr

**Keywords:** peroxisome-proliferator-activated receptor, fatty acid oxidation, doping control, regulatory T cells, inflammation, exercise

## Abstract

Synthetic ligands of peroxisome-proliferator-activated receptor beta/delta (PPARβ/δ) are being used as performance-enhancing drugs by athletes. Since we previously showed that PPARβ/δ activation affects T cell biology, we wanted to investigate whether a specific blood T cell signature could be employed as a method to detect the use of PPARβ/δ agonists. We analyzed in primary human T cells the in vitro effect of PPARβ/δ activation on fatty acid oxidation (FAO) and on their differentiation into regulatory T cells (Tregs). Furthermore, we conducted studies in mice assigned to groups according to an 8-week exercise training program and/or a 6-week treatment with 3 mg/kg/day of GW0742, a PPARβ/δ agonist, in order to (1) determine the immune impact of the treatment on secondary lymphoid organs and to (2) validate a blood signature. Our results show that PPARβ/δ activation increases FAO potential in human and mouse T cells and mouse secondary lymphoid organs. This was accompanied by increased Treg polarization of human primary T cells. Moreover, Treg prevalence in mouse lymph nodes was increased when PPARβ/δ activation was combined with exercise training. Lastly, PPARβ/δ activation increased FAO potential in mouse blood T cells. Unfortunately, this signature was masked by training in mice. In conclusion, beyond the fact that it is unlikely that this signature could be used as a doping-control strategy, our results suggest that the use of PPARβ/δ agonists could have potential detrimental immune effects that may not be detectable in blood samples.

## 1. Introduction

The nuclear receptor peroxisome-proliferator-activated receptor beta/delta (PPARβ/δ) plays an important role in muscle physiology [1]. This transcription factor can be activated by endogenous natural ligands, such as certain lipid metabolites, or synthetic ligands, such as GW501516 and GW0742 [1]. The latter substances have also been called “exercise pills” or “exercise mimetics”, since they were shown to affect the expression of endurance-related genes and metabolic pathways leading to increased exercise endurance [2]. While synthetic PPARβ/δ agonists so far have not been approved for clinical purposes for treating diseases such as dyslipidemia due to the discovery of carcinogenic properties in preclinical studies on animals, these substances are being abused for performance-enhancing purposes in both humans and horses [3,4]. Therefore, since 2009, the list of prohibited substances and methods of doping, as established by the World Anti-Doping Agency, includes PPARβ/δ agonists. Methods to detect PPARβ/δ agonists are mostly focused on GW501516, and tests were developed for both blood and urine samples [5]. However, the emergence of new substances of this class means that new methods need to be developed that will allow the detection of any PPARβ/δ agonist. One method could be to identify a blood signature that would be specific for PPARβ/δ activation. In this respect, our laboratory has previously published several studies demonstrating that PPARβ/δ activation induces significant immunometabolic changes in T cells. We showed that in vitro and in vivo treatment with GW0742 led to an increase in the mRNA levels of three genes involved in fatty acid oxidation (i.e., *Acaa2*, *Acadvl*, and *Cpt1a*) in isolated mouse primary T cells and lymph nodes, respectively, resulting in increased fatty acid oxidation (FAO) in these cells [6]. Furthermore, increased PPARβ/δ activity had an impact on T cell development in the thymus, resulting in reduced production of αβ-T cells, while γδ-T cell production was unaffected. This led to a decrease in the αβ/γδ T cell ratio in peripheral tissues, including blood.

Regulatory T cells (Tregs) are a subset of T cells important for maintaining self-tolerance by downregulating the immune response, and they do so by secreting immunoregulatory cytokines, such as TGF-β and IL-10, which act to suppress the activity and function of immune effector cells (e.g., CD4+ and CD8+ T cells, monocytes/macrophages, natural killer cells, and dendritic cells) [7]. While mouse Tregs have a certain flexibility in metabolic fuel choice, they have a preference for FAO [8]. It was therefore not unexpected, given our above-mentioned result showing an increased FAO in T cells following activation of PPARβ/δ, that we observed an increased prevalence of CD4+FOXP3+ Tregs in mouse lymph nodes after in vivo GW0742 treatment [9].

Whether these observed PPARβ/δ-induced changes in T cell parameters can be used as a blood signature for detection of the use of PPARβ/δ agonists depends on whether these changes are specific to PPARβ/δ activation and are not also potentially induced by other factors such as acute or chronic exercise. Exercise-induced immune changes have been described previously [10]. Many of them affect the Treg population or the ability of T cells to produce pro/anti-inflammatory cytokines [10,11]. However, the ability of physical fitness or exercise to directly modify the metabolism of immune cells is unproven [12] but could be involved in these changes.

Our objective in this study was to confirm, in human T cells, our previous published observations in mouse T cells, that PPARβ/δ activation leads to an increase in FAO. Likewise, we also wanted to determine the effect of PPARβ/δ activation on the induction of Treg polarization in human T cells. Furthermore, we investigated whether exercise training would interfere with the effects of PPARβ/δ activation on FAO gene expression, T cell ratios, and Treg polarization.

## 2. Results

### 2.1. In Vitro Treatment of Human T Cells with GW0742 Increases Their FAO Potential and Polarization in Tregs

**Figure 1:**
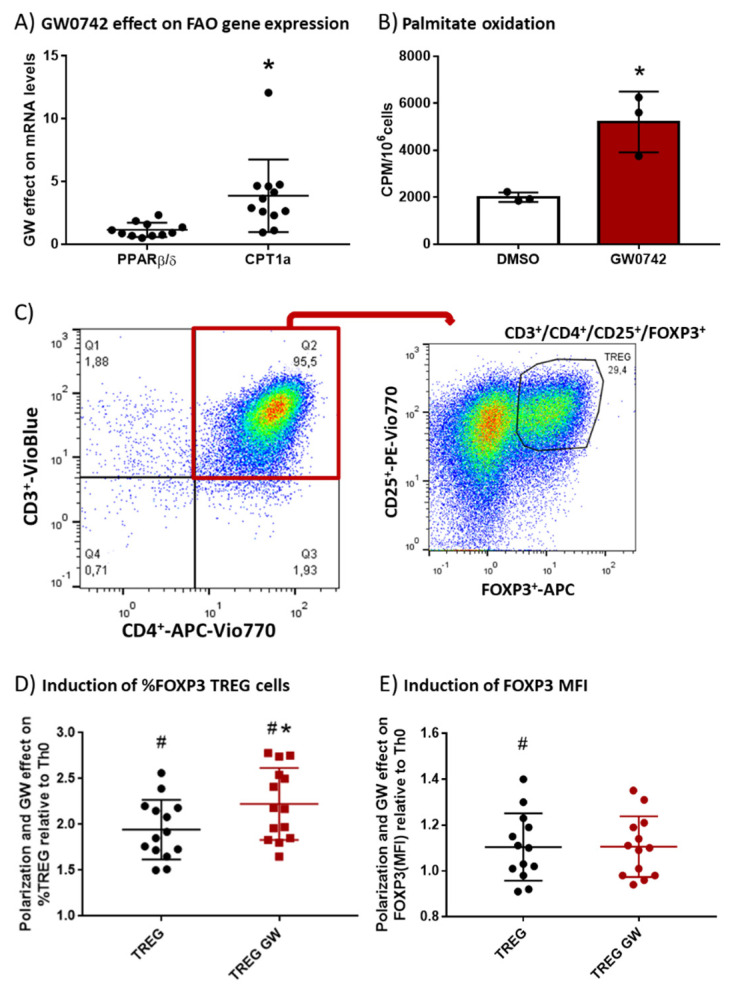
In vitro treatment of human T cells with GW0742 increases their FAO potential and their polarization in Tregs. (**A**) GW0742 1 µM effect (compared to DMSO, *n* = 8) on PPARβ/δ and CPT1a mRNA level reported to RPL27 mRNA level used as housekeeping mRNA on monocyte-depleted human buffy coats activated with αCD3 and αCD28 antibody-coated beads and cultured for 6 days with human IL-2 (20 ng/mL). (**B**) Palmitate oxidation in isolated human CD4+ T cells. FAO was measured as ^3^H-palmitate conversion to ^3^H_2_O and quantified as CPM/10^6^ cells in in vitro–activated CD4+ cells treated with 1 µM GW0742 or DMSO (*n* = 3). (**C**) Gating strategy of flow cytometry analysis of CD25+ FOXP3+ cells (Tregs) in CD3+CD4+ human T cells. (**D**) Fold induction of prevalence (%) of CD25+ FOXP3+ cells (Tregs) in enriched CD4+ T cells (*n* = 14) derived from monocyte-depleted human buffy coats treated in vitro with TGF-β (5 µg/mL) to induce Treg polarization in the presence of DMSO (TREG) or 1 µM GW0742 (TREG GW) relative to Th0 cells (nonpolarized cells). (**E**) Fold induction of FOXP3+ MFI (mean fluorescent intensity) in CD4+ T cells (*n* = 14) derived from monocyte-depleted human buffy coats treated in vitro with TGF-β (5 µg/mL) to induce Treg polarization in the presence of DMSO (TREG) or 1 µM GW0742 (TREG GW) relative to Th0 cells. Data are shown as mean ± SD. * *p* < 0.05, GW effect; # *p* < 0.05, TREG vs. Th0 cells (univariate *t*-test).

We hypothesized that GW0742, a PPARβ/δ agonist, preconditions human T cells and favors their polarization toward the Treg subtype. To validate this hypothesis, we isolated PBMCs (peripheral blood mononuclear cells) from human buffy coats and performed monocyte depletion by adhesion. The remaining cells, enriched in lymphocytes, were placed in culture, activated with beads coated with αCD3 and αCD28 antibodies and IL-2, and treated with 1 µM GW0742 or left untreated for 6 days. We showed (Figure 1A) a significant fourfold induction by GW0742 treatment of carnitine palmitoyl transferase 1a (CPT1a) mRNA that encodes the enzyme limiting the entry of fatty acids into the mitochondria, leading to a 2.6-fold increase in palmitate oxidation (Figure 1B). However, no difference in PPARβ/δ mRNA level was observed (Figure 1A). As already demonstrated in mouse T cells from secondary lymphoid organs [6], activation of the PPARβ/δ pathway in human blood T cells induces the expression of genes encoding FAO proteins (CPT1a) and increases FAO. As Treg cells are very dependent on FAO, we studied the impact of PPARβ/δ pathway activation on human T cell polarization toward Tregs. In this objective, we cultured CD4+ T cells selected from monocyte-depleted human buffy coats and treated in vitro with TGF-β (5 µg/mL) to induce Treg polarization in the presence of DMSO (TREG) or GW0742 (TREG GW). We used the gating strategy presented in Figure 1C, namely a flow cytometry analysis of CD25+ FOXP3+ cells (Tregs) in CD3+CD4+ human T cells. The presence of TGF-β in the culture medium of CD4+ T cells (Figure 1D, TREG) permitted to almost double (1.94 ± 0.09, # *p* < 0.0001) the percentage of CD25+ FOXP3+ cells (Tregs) in CD3+CD4+ human T cells compared to the condition without TGF-β (Th0) and induced a slight but significant increase in FOXP3 mean fluorescent intensity (MFI) (Figure 1E, TREG, 1.104 ± 0.04, # *p* < 0.05), considered to represent the mean content level of FOXP3 protein in cells. The activation of the PPARβ/δ pathway by GW0742 (Figure 1D, TREG GW) significantly favored Treg polarization in 14 independent experiments, as reflected by the increase in prevalence of CD25+ FOXP3+ cells in CD3+CD4+ human T cells (mean increase 14.9% ± 3.1%) without change in FOXP3 MFI. We, therefore, showed that activation of PPARβ/δ pathway in human blood T cells leads to a change in T cell metabolism, favoring FAO that is accompanied by an increase in Treg polarization. These changes could perhaps be used as a blood signature of the abuse of PPARβ/δ agonists by athletes.

### 2.2. GW0742 Treatment Increases FAO Potential and Leads to Differential Changes in Treg Prevalence in Mouse Secondary Lymphoid Organs Depending on Training Status

**Figure 2:**
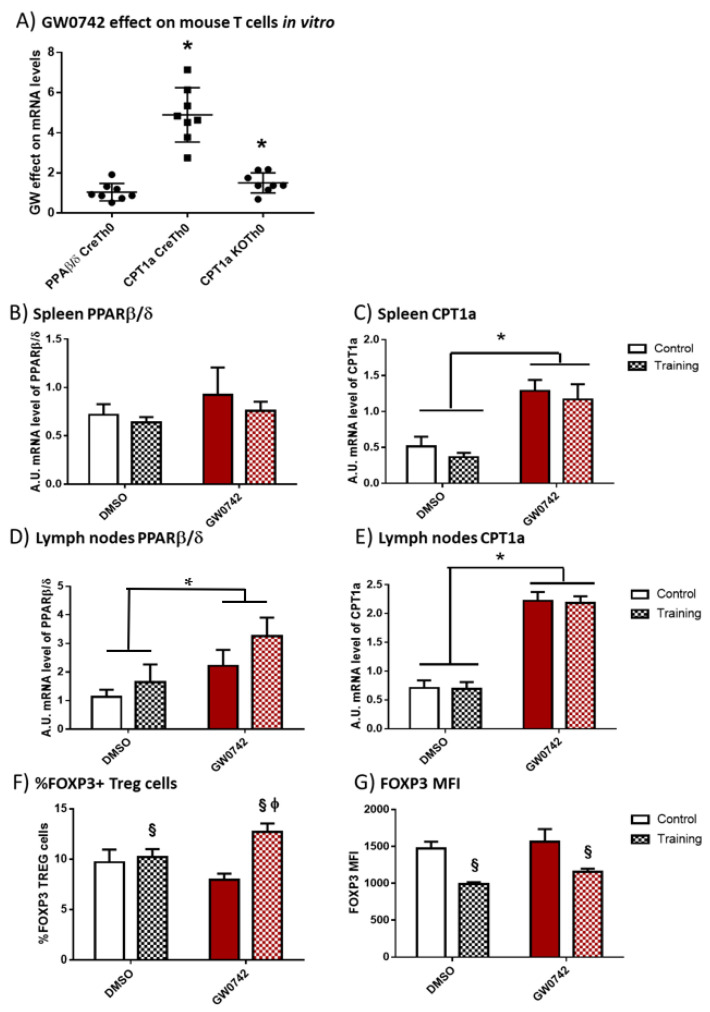
In vitro treatment of mouse T cells with GW0742 increases their FAO potential, and in vivo GW0742 treatment of mice leads to differential changes in FAO potential and Treg profile in the lymph nodes and spleen in trained mice. (**A**) The effect of 1 µM GW0742 (compared to that of DMSO, *n* = 6) on PPARβ/δ and CPT1a mRNA levels normalized to 36B4 mRNA level used as housekeeping mRNA in CD4+ T cells from Lck-Cre (Cre) or KO-T-PPARβ/δ mice (KO) activated with αCD3 and αCD28 antibodies coated-beads and cultured for 6 days with mouse IL-2 (20 ng/mL). (**B**) PPARβ/δ mRNA level and (**C**) CPT1a mRNA level in spleen; (**D**) PPARβ/δ mRNA level and (**E**) CPT1a mRNA level in lymph nodes, from control or trained mice (8 weeks, *n* = 6 per group) treated, or not treated, for 6 weeks with GW0742 (3 mg/kg BW/day). (**F**) Prevalence (%) of FOXP3+ cells (Tregs) in cells extracted from lymph nodes from control or trained mice (8 weeks, *n* = 6 per group) treated, or not treated, for 6 weeks with GW0742 (3 mg/kg BW/day). (**G**) FOXP3+ MFI (mean fluorescent intensity) in cells extracted from lymph nodes from control or trained mice (8 weeks, *n* = 6 per group) treated, or not treated, for 6 weeks with GW0742 (3 mg/kg BW/day). Data are shown as mean ± SD. * *p* < 0.05, GW0742 effect; § *p* < 0.05, training effect; and ϕ *p* < 0.05, interaction effect between training and GW0742 (two-way ANOVA).

We first validated whether the potential signature that was detectable in human cells (i.e., increased FAO potential and Treg polarization) is specific for PPARβ/δ activation. We have previously demonstrated [6] that treatment of murine T cells with GW0742 increased palmitate oxidation and this effect was lost when the cells were co-treated with etomoxir, an inhibitor of CPT1a. We isolated CD4+ T cells from secondary lymphoid organs (SLO) of controls (Cre) or mice invalidated for PPARβ/δ in T cells (KO-T-PPARβ/δ), treated the cells with 1 µM of GW0742 for 6 days and studied the consequences on PPARβ/δ and CPT1a mRNA level. We showed, as seen in Figure 2A, that the treatment of control cells (CreTh0) with GW0742 did not alter the mRNA level of PPARβ/δ. However, GW0742 treatment increased the mRNA level of CPT1a by a factor of 5. This GW0742 effect seemed specific to its action on PPARβ/δ, since the induction of CPT1a mRNA was markedly reduced in cells isolated from KO-T-PPARβ/δ mice (KOTh0).

To discriminate between the effects induced by chronic (training) bouts of exercise and PPARβ/δ activation, mice were trained on treadmills for 8 weeks and they were, or were not, treated with GW0742 mixed with food for 6 weeks (3 mg/kg BW/day). At the end, the spleen and lymph nodes were harvested. GW0742 treatment led to an increase in PPARβ/δ mRNA levels in the lymph nodes but not in the spleen (Figure 2B,D). Moreover, the treatment of control mice with GW0742 for 6 weeks induced a significant increase in CPT1a mRNA by a factor of 3.3 ± 1.5 in the spleen (Figure 2C) and 3.8 ± 1.7 in the lymph nodes (Figure 2E). We did not detect a significant effect of training on PPARβ/δ or CPT1a mRNA levels in the SLO, nor did training alter the effects of GW0742 (Figure 2B–E). Regarding Treg prevalence, GW0742 treatment of mice did not significantly affect the percentage of FOXP3+ T cells (Tregs) in the lymph nodes of mice (Figure 2F), but exercise training independently of GW0742 treatment significantly increased this percentage and decreased the MFI level of FOXP3 (Figure 2F,G). However, there was a significant combined effect of GW0742 treatment and training, significantly increasing the proportion of Treg cells in lymph nodes (Figure 2F). It is noteworthy that the GW0742 treatment did not affect the FOXP3 MFI level of trained mice (Figure 2G). Thus, the GW0742 treatment of mice leads to immunometabolic changes promoting FAO potential and an increased proportion of Treg cells in exercise-trained mouse secondary lymphoid organs. 

### 2.3. The Detection of GW0742 Effect on FAO Potential Is Masked in the Blood of Trained Mice

**Figure 3:**
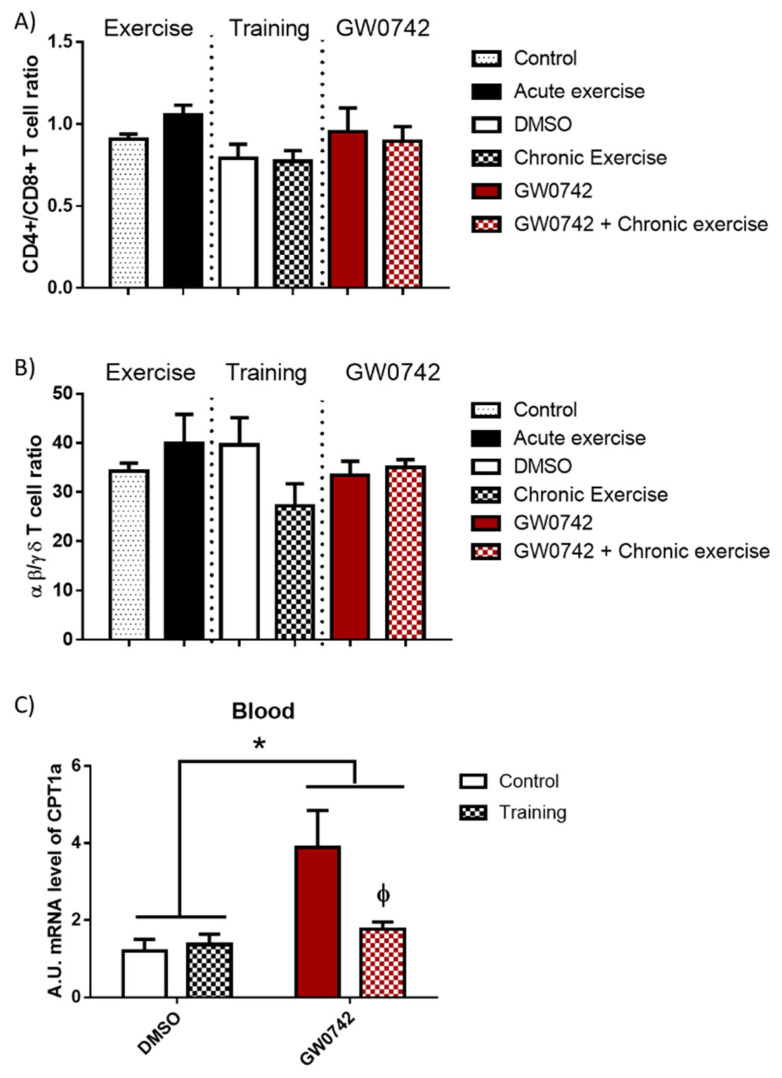
The T cell profile (CD4+/CD8+ T cell ratio, αβ/γδ T cell ratio) is unchanged in the blood by GW0742 treatment, exercise, or training. However, the detection in whole blood of GW0742’s effects on the FAO potential is reduced by training. Mice were either subjected to or not given (control, *n* = 10) acute exercise on a treadmill with a slope of 5° (*n* = 8), the speed of the treadmill increased by 5 cm/s every 15 min until mouse exhaustion. Another cohort of mice (*n* = 6 per group) was trained (chronic exercise) on a treadmill for 8 weeks, or not trained, and were then treated, or not treated (given DMSO instead), for 6 weeks with GW0742 (3 mg/kg BW/day). Blood mononuclear cells were isolated using Ficoll gradient, stained with fluorescence-conjugated antibodies, and analyzed with a BD FACS Canto II flow cytometer. (**A**) The CD4+/CD8+ T cell ratio was calculated; (**B**) the αβ/γδ T cell ratio was calculated; (**C**) the CPT1a mRNA level in blood cells was normalized by 36B4. Data are shown as mean ± SD. * *p* < 0.05, GW0742 effect, and ϕ *p* < 0.05, interaction effect between training and GW0742 (two-way ANOVA).

In mice, we showed that increased PPARβ/δ activity leads to a defect in T cell development in the thymus with subsequent consequences on T cell populations in peripheral lymphoid organs, characterized by a decrease in the αβ/γδ T cell ratio. This was accompanied by an increase in FAO potential and a concomitant increase in CPT1a mRNA levels in lymphoid organs [6,9]. To definitively validate this signature of increased PPARβ/δ activity, which could be detected in athletes’ blood, its effects must be discriminated from those induced by acute and chronic (training) bouts of exhaustive exercise. We submitted mice to acute exercise, training (8 weeks), or long-term treatment (6 weeks) with GW0742 and studied the evolution in the blood of some signature markers (αβ/γδ T cell ratio, CPT1a mRNA levels). We also wanted to check whether the signature of the GW0742 use could be distinguished from that of physical training. We showed that the CD4+/CD8+ T cell ratio (Figure 3A) and the αβ/γδ T cell ratio (Figure 3B) were not altered in the blood either by acute exercise, training, and GW0742 treatment, or by the combination of both GW0742 and training. We found (Figure 3C) that mRNA levels of CPT1a in the blood were not impaired by training but, in contrast, were significantly largely increased by treatment with GW0742 (4.04 ± 3.03-fold increase). This measurement in blood cells of the CPT1a mRNA levels could, thus, constitute a signature of the use of the GW0742. However, and very surprisingly, we can see that the effect of GW0742 on CPT1a mRNA levels was largely and significantly decreased when GW0742 intake was combined with endurance training. Thus, the signature of the use of GW0742 is masked by endurance training, and the measurement of blood cell CPT1a mRNA levels will not be a reliable marker for the use of GW0742.

## 3. Discussion

T cells’ function is intimately linked to their metabolic programs [13,14]. While Tregs rely heavily on FAO, they have found ways to adapt to different tissue types, such as tumors, to survive in competitive environments [15]. Mouse Treg cells generated through in vitro polarization of CD4^+^ T cells preferentially use FAO [16,17]. However, it is still controversial whether human Treg cell differentiation is dependent on FAO. We show here that the use of substances that activate PPARβ/δ can increase FAO in human T cells in vitro, and as a result increase the prevalence of Tregs. This result is important and new. Human Tregs are metabolically distinct from their mouse counterparts. It is known that ex vivo human Tregs are more glycolytic than ex vivo mouse Tregs [18]. This baseline difference may account for the magnitude of detectable metabolic changes that could be induced by either an endogenous or exogenous modulator of PPARβ/δ activity. Thus, we can assume that the whole-body GW0742 effect on immunometabolism would be more potent in humans compared to that in mice. Since our in vivo studies were conducted in mice, it is plausible that the effects observed in mice will be stronger in humans.

Whether exercise can modulate immune function by metabolic changes remains an underexplored area of research, and the ability of physical fitness or exercise to directly modify the metabolism of immune cells is unproven [12]. In obese mice, metabolic changes induced by exercise training were characterized by an increase in AMPK activity, both in lymphoid tissues and in skeletal muscle [9]. In both tissues, GW0742 treatment had complementary effects to exercise training on the decrease in inflammatory markers [9]. In the present study, in secondary lymphoid tissues, the induction of CPT1a expression was independent of exercise and was characteristic of the GW0742 effect on increasing FAO potential. The magnitude of CPT1a induction was high and suggests that the metabolism of immune cells (mainly T cells) was altered by GW0742 treatment. Furthermore, PPARβ/δ expression was also increased in lymph nodes by GW0742 treatment. However, the prevalence of Tregs was unchanged in the lymph nodes of sedentary mice treated with GW0742. Therefore, we can conclude that, even though GW0742 increased PPARβ/δ and CPT1a expression, it did not increase Treg prevalence in mice lymph nodes. Exercise training significantly decreased the MFI level of FOXP3 but interacted with GW0742, leading to an increase in the prevalence of Tregs. This increase appeared despite an absence of effect on CPT1a mRNA level in secondary lymphoid tissues. Together, these findings suggest that, at least in mice, CPT1a expression levels are disconnected from Treg prevalence. These results are entirely in line with the work of Raud et al. [19] that showed, using a mouse genetic model in which CPT1a was abrogated in T cells, that the ACC2/CPT1a axis is dispensable for Treg cell formation.

Despite an absence of effect on CPT1a mRNA level of exercise in SLOs, it is known that exercise impairs aspects of cellular immune function, probably due to the higher energy cost of exercise and metabolic perturbations in endurance athletes [20]. Indeed, a rapid metabolite turnover can be detected in seconds after an acute bout of endurance exercise, whereas it takes minutes to hours for transcriptomic and proteomic responses accounting for training adaptation [21]. An increase of about 75% of the maximum rate of fat oxidation (whole body measure), which is already high in elite endurance athletes, is observed after a 2 h recovery in a fasting condition from an endurance exercise session [22,23,24]. As both exercise and GW0742 alter fatty acid availability, we considered it important to choose the most appropriate experimental conditions in mice that allowed discriminating GW0742 effects from those induced by exercise, considering that the signature of an increase in FAO in T cells would be interpreted in an individual athletes’ biological passport [25] as a doping signature. Information is available on the internet regarding the oral doses of GW0742 used by athletes for the purpose of doping. The oral dosages used comprise between 10 to 50 mg per day for 4 to 8 weeks, which in terms of availability would correspond to a dose of 1–10 mg/kg administered in mice. Notably, the plasma concentration of the ligand at the 1 mg/kg dose in mice is shown to specifically activate PPARβ/δ [26]. We used a dosage/treatment period in our mouse studies that is quite close to the doping protocol used by athletes by administering a dose of 3 mg/kg persistently in food for 6 weeks. We used blood samples from trained mice to detect interactions between GW0742 and exercise training effects. GW0742 induced an increase in CPT1a mRNA, but surprisingly, this induction was masked by the training status of mice. This questioned the relevance of this signature for doping-control strategies. Another suggested alternative is based on our previous study that proposed the αβ/γδ T cell ratio as a T cell signature to detect activity of the PPARβ/δ pathway [6]. We showed here that neither acute or chronic exercise nor GW0742 treatment changed this αβ/γδ T cell ratio in mouse blood. Perhaps the 6-week GW0742 treatment was not long enough for alterations in T cell development in the thymus to be reflected in the blood (our previous study examined transgenic mice that overexpressed PPARβ/δ in T cells constitutively).

Outside of the potential to use the latter observations to develop novel methods to detect the use of substances that activate the PPARβ/δ pathway, it should be noted that these novel discoveries also suggest that athletes who take PPARβ/δ agonists might seriously disturb their T cell homeostasis, thereby endangering the effectiveness of their immune system. Forcing FAO in CD4+ T cells would result in an increase in metabolic inflexibility. Depending on the (patho)physiological context, this could have either beneficial or deleterious consequences. The immunomodulatory effects of exercise might be mediated by the ability of exercise to adjust and improve Treg number and function [27]. An increase in Tregs would augment immune tolerance, thereby decreasing the risk of development of autoimmune diseases [28]. It should be noted in this context that physical exercise is known to decrease the risk of developing and is beneficial to the management of autoimmune disease [29]. Adipose tissue Tregs have been shown to play a beneficial role in decreasing insulin resistance associated with diet-induced obesity but a deleterious role in age-associated insulin resistance [30,31]. In the context of cancer, Tregs suppress anticancer immunity and, by doing so, hinder protective immunosurveillance of tumors and hamper effective antitumor immune responses [32]. A recent publication demonstrated that PPARβ/δ plays an important role in Treg survival and function in tumors [33]. It was observed that intratumoral Tregs displayed increased expression of multiple PPARβ/δ target genes compared to Tregs from spleen and lymph nodes. Knocking out PPARβ/δ specifically in Tregs led to a reduction in intratumoral Treg accumulation accompanied by decreased tumor growth. Taken together, these data suggest that there is a real possibility that abuse of PPARβ/δ agonists for performance-enhancing purposes might lead to an increased cancer risk and/or a worse outcome when a tumor develops.

To conclude, we show here that the use of substances that activate PPARβ/δ can increase FAO in human T cells and as a result increase the prevalence of Tregs. It is unlikely that this signature could be used as a doping-control strategy in athlete’s blood, since these immunometabolic changes are masked in mice by training status. Moreover, our study alerts on the risks of immune surveillance alterations with the use of PPARβ/δ activators in order to improve physical performance.

## 4. Materials and Methods

### 4.1. Animal Experiments

#### 4.1.1. Acute Treadmill Exercise

Twelve-week-old wild-type mice (*n* = 18) purchased from Charles River (Ecully, France) were accustomed to the treadmill (five-lane motorized treadmill, LE8710 M, Bioseb) a week before the running test was performed with a slope of 5° (*n* = 18). During a warm-up phase, the speed of the treadmill was progressively increased every 2 min for 10 min (5 to 25 cm/s). This phase was followed by an acute exercise phase where the speed of the treadmill was increased by 5 cm/s every 15 min (30 to 40 cm/s) until the mice exhibited signs of exhaustion. The rear of the treadmill was equipped with a low-voltage electric stimulating bar to encourage each mouse to run. The bar was set to deliver 0.2 mA at a frequency of 0.25 Hz, which caused an uncomfortable shock but did not injure the animal. The number of shocks was recorded, and the electric delivery was stopped if 50 shocks were reached.

#### 4.1.2. Physical Training and GW0742 Treatment of Mice

**Figure 4:**
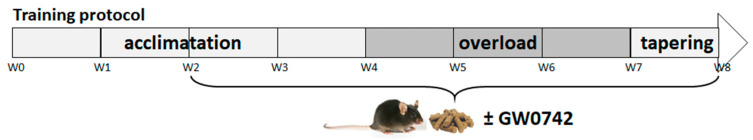
Training and GW0742 mouse treatment procedure.

We used 7-week-old C57Bl/6J wild-type mice purchased from Charles River (Ecully, France). Animals were maintained in a 12 h light, 12 h dark cycle and received food (A04 from UAR (Usine d’Alimentation Rationnelle), Villemoisson sur Orge, France) and water ad libitum. The mice were trained (8 weeks) on the five-lane treadmill. The training protocol was divided into three phases (Figure 4). The acclimatation phase lasted 4 weeks, during which the mice were trained in three sessions per week. The overload phase lasted 3 weeks, during which the mice were trained in five sessions per week. Finally, the tapering phase lasted 1 week, during which the mice were trained in three sessions. A training session lasted between 20 and 40 min, the treadmill speed varied between 20 and 40 cm/s, and the belt was positively inclined at 5°. To encourage the mice to run, electrical (0.2 mA–160 kΩ) and mechanical stimulation were used. 

After 2 weeks of acclimatation to training, mice received a normal chow diet (standard chow diet (A04)) administered ad libitum, supplemented with GW0742 (3 mg/kg BW/day) or with the vehicle (dimethyl sulfoxide, DMSO, 1%). Food was reconstituted as described previously [9,34]. Twice a week, the food was refreshed, and animals were weighed.

Animals were sacrificed (90 min after acute exercise or 24 h after the last training session) by a lethal dose of intraperitoneal ketamine/xylazine (100/16 mg/kg). Blood samples were obtained by cardiac puncture.

### 4.2. Mouse and Human T Cell Isolation and Treg Polarization

Mouse CD4+ cells from control (Lck-Cre) or KO-T-PPARβ/δ mice [35] were positively selected from secondary lymphoid organs (SLOs, consisting of the inguinal, brachial, and cervical lymph nodes and the spleen). CD4+ T cells were grown as previously described [36] at 4 × 10^5^ cells/well in a 48-well plate in RPMI medium supplemented with 1 mM sodium pyruvate, nonessential amino-acid (1×), 1% penicillin/streptomycin, 10% fetal calf serum (FCS), and 50 µM β-mercaptoethanol. Activation beads, covalently bound to αCD3 and αCD28 antibodies, as well as mouse IL-2 (20 ng/mL), were added to the culture medium for the Th0 conditions, as well as PPARβ/δ agonist GW0742 1 µM or DMSO (0.1%). Cell medium was complemented with an equal volume of fresh medium every three days.

Human buffy coats from healthy donors (Établissement Français du Sang, Marseille, France) were used to collect PBMCs by Ficoll density gradient centrifugation. Monocytes were depleted by adherence to Primaria plates for 2 h. The cells of the supernatant (enriched in T cells), or CD4+ T cells isolated by negative selection using a Miltenyi Biotec system (#130-091-155), were plated at 4 × 10^5^ cells/well in a 48-well plate. Activation beads coated with αCD3 and αCD28 antibodies, as well as human IL-2 (20 ng/mL), were added to the culture medium in presence or absence of GW0742 (1 µM) or DMSO (0.1%). After 6 days of activation, the cultures comprised over 95% T cells (data not shown). For Treg polarization experiments, TGF-β (5 µg/mL) was added to the culture medium for 6 days.

### 4.3. Measurement of β-Oxidation Using ^3^H-Labeled Palmitate

The isolated human CD4+ cells were cultured at 4 × 10^5^ cells/well in a 48-well plate in RPMI and were activated with anti-CD3/anti-CD28-coated beads. The palmitate β-oxidation was evaluated as previously described in [6,35,36]. Briefly, after 5 days of activation, for the last 24 h, we added in the wells a mix of radioactive and nonradioactive palmitate coupled to BSA (2:1 ratio; 30 μM Na-palmitate, 15 μM fatty-acid-free BSA and 10 μCi (0.83 μM 9,10-^3^H-palmitic acid (Perkin Elmer)). After a 24 h additional incubation, 100% trichloroacetic acid (10% final) was added to the cell suspensions, and proteins were precipitated. After centrifugation, NaOH (final concentration 0.75 M) was added to the supernatant to increase pH to 12. Subsequently, 400 μL of supernatant was applied to ion-exchange columns (Dowex 1 × 2–400 resin), and ^3^H_2_O was recovered by eluting with 4 mL of H_2_O. A 0.75 mL aliquot was then used for scintillation counting. Results were expressed as CPM (counts per minute) per 10^6^ cells.

### 4.4. Cell Preparation and Flow Cytometry Analysis

Human CD4+ in culture, mouse lymph node cell suspension (1 million), or blood mononuclear cells isolated using Ficoll gradient were stained with fluorescence-conjugated antibodies (αCD3-VioBlue or αCD3-FITC, αCD4-APC-Vio770 or αCD4-APC, αCD25-PE-Vio, αFOXP3-APC, αTCRβ-PEcy7, αTCRγδ-PE), and stained cells were analyzed with a BD FACS Canto II flow cytometer (BD Biosciences, Franklin Lakes, NJ, USA) using Miltenyi Biotec (Paris, France) antibodies. The extracellular labeling (αCD3, αCD4, αTCRβ, αTCRγ, and αCD25) was done at 4 °C for 20 min. After two washes with PBS 0.5% FCS plus 2 mM EDTA, cells were permeabilized and fixed following the manufacturer’s protocol (Miltenyi Biotec Kit). The intracellular staining was performed with αFOXP3 after the extracellular labeling. To determine the percentage of Tregs in a cell population, we discriminated CD3+CD4+ cells, and in this population, we gated FOXP3+ cells for mice cells and CD25+FOXP3+ cells for human cells, as shown in Figure 1C. Data were analyzed using FlowJo software.

### 4.5. RNA Extraction and Quantitative Real-Time PCR

Total RNA was extracted from cells or tissues with Trizol reagent (Invitrogen). For isolating RNA from blood, we used the kit Mouse RiboPure–Blood RNA Isolation (Applied Biosystems) following manufacturer procedure. Then, 1 μg of RNA was reverse-transcribed using a QuantiTect Reverse Transcription Kit (Qiagen) on a Q-cycler II. Quantitative PCR was done using SYBR Premix Ex Taq (Tli RNase H Plus) (Ozyme) on a StepOne machine (Life Technologies). The relative amount of all mRNAs was calculated using the comparative ΔΔCT method, and either 36B4 (for mice) or RPL27 (for humans) was used as the housekeeping gene. Primer sequences are available upon request.

### 4.6. Statistical Analyses

For each dependent variable under consideration, and according to assumptions for statistical analysis (i.e., normal distribution, equal variance), we performed the following: (1) nonparametric Mann–Whitney (in vitro study); (2) one-way ANOVA analysis (acute exercise effect); (3) two-way ANOVA analyses to investigate independent effects of GW0742 treatment and exercise training, and the interaction effects between GW0742 and training. Statistical significance was accepted at *p* < 0.05. The results are presented as means ± standard deviations. All data were analyzed using StatView and GraphPad Prism v 5.0 software (San Diego, CA, USA).

## Data Availability

The data presented in this study are available on request from the corresponding author.
